# Luminal Localization of α-tubulin K40 Acetylation by Cryo-EM Analysis of Fab-Labeled Microtubules

**DOI:** 10.1371/journal.pone.0048204

**Published:** 2012-10-26

**Authors:** Virupakshi Soppina, Jeffrey F. Herbstman, Georgios Skiniotis, Kristen J. Verhey

**Affiliations:** 1 Department of Cell and Developmental Biology, University of Michigan Medical School, Ann Arbor, Michigan, United States of America; 2 Life Sciences Institute and Department of Biological Chemistry, University of Michigan Medical School, Ann Arbor, Michigan, United States of America; University of Heidelberg Medical School, Germany

## Abstract

The αβ-tubulin subunits of microtubules can undergo a variety of evolutionarily-conserved post-translational modifications (PTMs) that provide functional specialization to subsets of cellular microtubules. Acetylation of α-tubulin residue Lysine-40 (K40) has been correlated with increased microtubule stability, intracellular transport, and ciliary assembly, yet a mechanistic understanding of how acetylation influences these events is lacking. Using the anti-acetylated tubulin antibody 6-11B-1 and electron cryo-microscopy, we demonstrate that the K40 acetylation site is located inside the microtubule lumen and thus cannot directly influence events on the microtubule surface, including kinesin-1 binding. Surprisingly, the monoclonal 6-11B-1 antibody recognizes both acetylated and deacetylated microtubules. These results suggest that acetylation induces structural changes in the K40-containing loop that could have important functional consequences on microtubule stability, bending, and subunit interactions. This work has important implications for acetylation and deacetylation reaction mechanisms as well as for interpreting experiments based on 6-11B-1 labeling.

## Introduction

Microtubules are cytoskeletal filaments that play important roles in the organization, shape, motility and division of eukaryotic cells [1]. Microtubules consist of αβ-tubulin heterodimers that self-assemble head-to-tail to form protofilaments and laterally to form a hollow tube. The αβ-tubulin subunits can undergo a variety of evolutionarily-conserved post-translational modifications (PTMs) including acetylation, polyglutamylation, polyglycylation, detyrosination, phosphorylation and palmitoylation that are thought to regulate the polymerization properties of tubulins and/or their interactions with microtubule associated proteins (MAPs) and motor proteins. Thus, PTMs provide functional specialization to microtubules ranging from structural support to intracellular trafficking [2].

A prominent PTM of microtubules is the acetylation of the ε-amino group of Lysine-40 (K40) of α-tubulin [3], [4]. K40 acetylation has been widely noted due to the availability of a monoclonal antibody 6-11B-1 that binds to K40-acetylated α-tubulin across a wide variety of species [5]. K40 acetylation accumulates on a subset of cytoplasmic microtubules as well as microtubules in the spindle, axon and cilia. Despite its widespread occurrence, the functional significance of K40 acetylation remains unclear. Microtubule acetylation has been implicated in regulating a variety of cellular functions including ciliary assembly, intracellular trafficking, cell motility, and axon outgrowth [2], [6]. These effects may be due to direct effects of K40 acetylation on microtubule dynamics as acetylation is generally believed to mark “stable” microtubules (resistant to depolymerizing conditions), yet whether K40 acetylation directly influences microtubule dynamics is controversial [7]–[11]. K40 acetylation can influence interactions between neighboring αβ- tubulin subunits and thus affect protofilament number and organization in worms [12], [13]. Notably, K40 acetylation has been suggested to directly impact events on the surface of cellular microtubules such as severing [14] and the binding and motility of kinesin-1 and cytoplasmic dynein motors [15]–[18].

The K40 residue resides in a loop of α-tubulin that was found disordered in both the electron crystallographic structure of αβ-tubulin and a high resolution cryo-EM microtubule reconstruction, but is thought to be positioned in the lumen of the microtubule [19]–[21]. How a luminal residue becomes modified by cytoplasmic enzymes is not known. Also unclear is how acetylation of a luminal residue can affect the binding or function of motor proteins and MAPs on the surface of a microtubule. One possibility is that the K40-containing loop extends into the cytoplasm through the holes between tubulin subunits [20]. Indeed, a recent cryo-EM analysis of microtubules polymerized from GMPCPP (a non-hydrolyzable analogue of GTP)-tubulin indicates that the loop containing K40 lies near the pores between tubulin subunits and may be accessible from the outside of the microtubule [22]. Thus, the location and accessibility of the acetyl-K40 residue with respect to overall microtubule structure are important to define.

Understanding the molecular, structural, and functional consequences of α-tubulin K40 acetylation has been facilitated by the recent identification of the enzymes that acetylate and deacetylate this site *in vivo* and *in vitro*. Acetylation of K40 is carried out by MEC-17 [also known as α-tubulin acetyltransferase (αTAT)], a member of the GNAT family of lysine acetyltransferases [23]–[25]. Two enzymes have been identified that directly deacetylate K40 histone deacetylase 6 (HDAC6, a class IIb lysine deacetylase) and sirtuin2 (SIRT2, a class III lysine deacetylase) [8], [25]–[28]. In this study, we used MEC-17 and SIRT2 to generate acetylated and deacetylated tubulins, and then probed the location of the K40 acetylation site by electron cryo-microscopy (cryo-EM) and 3D reconstruction of 6-11B-1 Fab-labeled acetylated microtubules. We show definitively that the site of Fab attachment is in the lumen of the microtubule. Surprisingly however, the 6-11B-1 antibody recognizes both acetylated and deacetylated microtubules. In addition to the functional consequences of the K40 acetylation in the microtubule lumen, this work has important implications for interpreting experiments based on 6-11B-1 antibody labeling.

## Results

### Generation of highly acetylated or completely deacetylated tubulins

To determine the location of the K40 acetylation site on α-tubulin with respect to the overall microtubule architecture, we employed cryo-EM visualization and 3D reconstructions of highly acetylated microtubules decorated with Fab fragments derived from the monoclonal anti-acetylated tubulin (clone 6-11B-1) antibody. Since purified brain tubulin contains ∼40–50% K40-acetylated α-tubulin [29], we first set out to generate highly acetylated α-tubulin for maximum Fab occupancy and completely deacetylated α-tubulin for control experiments. For this purpose, we purified recombinant forms of the acetyltransferase MEC-17 and deacetylase SIRT2 enzymes (Figure S1A and B) as described [23], [24], [26].

**Figure 1:**
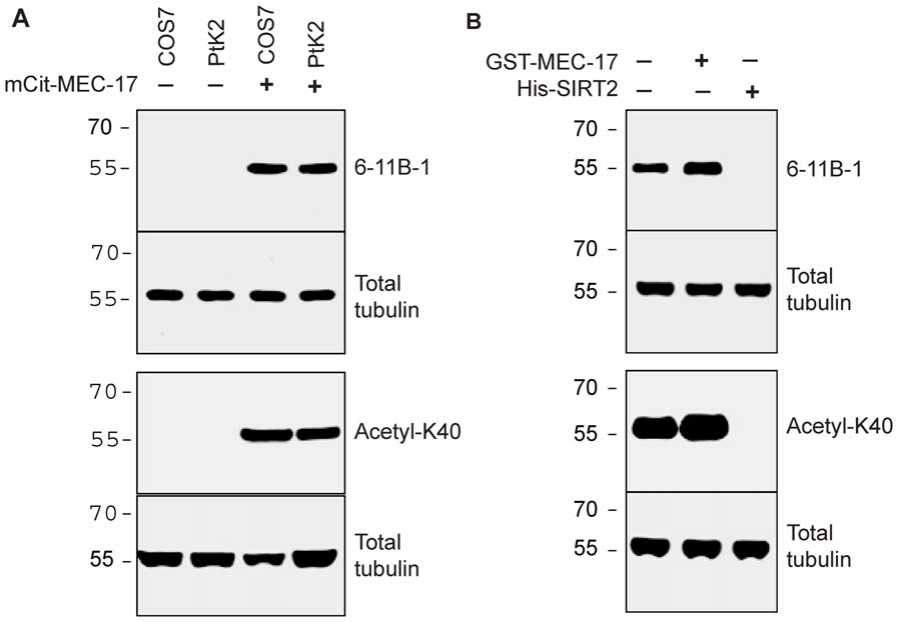
Generation of acetylated and deacetylated tubulins. **A**) Lysates of COS7 and PtK2 cells either untransfected (lanes 1 and 2) or expressing the acetyltransferase MEC-17 (lanes 3 and 4) were immunoblotted for K40 acetylation of α-tubulin using monoclonal 6-11B-1 and polyclonal anti-acetyl-K40 antibodies. **B**) Purified brain tubulin was untreated (lane 1) or treated with recombinant MEC-17 (lane 2) or SIRT2 (lane 3) enzymes. The total tubulin in all samples was determined in parallel by blotting with an anti-β-tubulin antibody.

To verify that these enzymes alter the acetylation state of the K40 residue, we performed western blotting with two antibodies. First, we used the monoclonal antibody 6-11B-1 that recognizes acetylated α-tubulin by immunostaining and western blotting [5]. Second, we used a rabbit polyclonal antibody (anti-acetyl-K40) raised against an acetylated peptide corresponding to the primary sequence of mouse α-tubulin. Both antibodies detected little to no acetylated α-tubulin in untransfected COS-7 or PtK2 cell lysates (Figure 1A, lanes 1 and 2) but detected a strong band of K40-acetylated tubulin in lysates from COS-7 and PtK2 cells expressing MEC-17 (Figure 1A, lanes 3 and 4), consistent with previous results [23], [24]. Note that acetylated α-tubulin can be detected in untransfected COS-7 lysates upon loading more material whereas untransfected PtK2 cells contain only unacetylated (never modified) α-tubulin despite the presence of the K40 residue in an α-tubulin sequence ([18] and data not shown). These results indicate that both antibodies specifically recognize the presence of acetyl-K40 in denatured α-tubulin.

**Figure 2:**
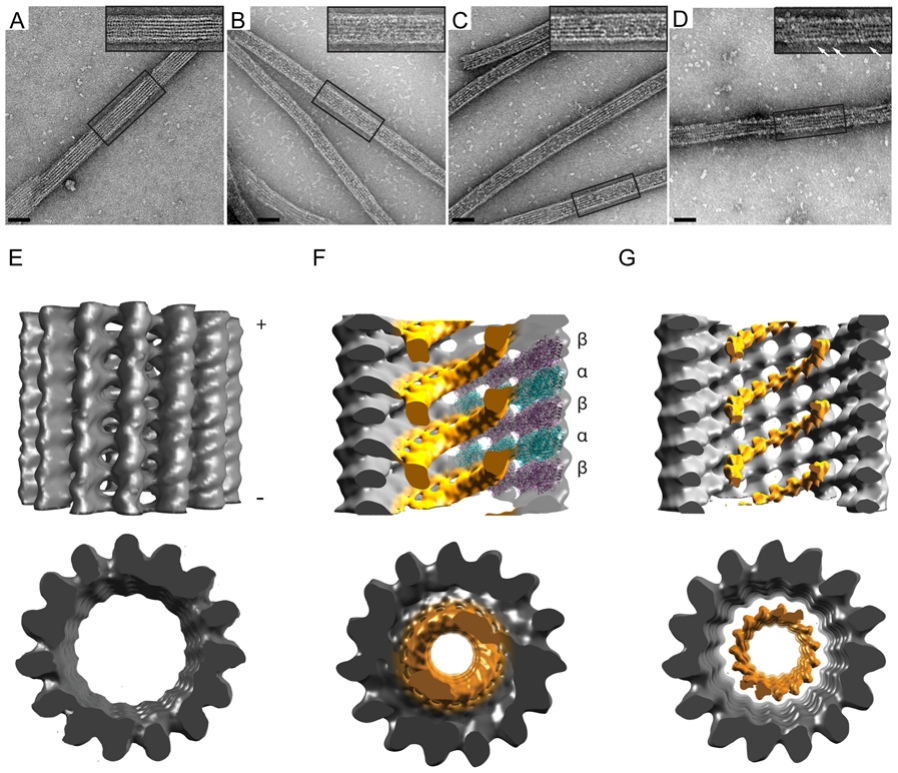
2D and 3D EM visualization of the 6-11B-1 Fab within the microtubule lumen. **A-D**) Microtubules polymerized from A,D) untreated B) MEC-17-treated (acetylated), or C) SIRT2-treated (deacetylated) tubulins were incubated with A-C) 6-11B-1 Fab fragments or D) GST-KHC motor domain and visualized after embedding in negative stain. The insets show expanded views of the boxed areas. White arrows in D) indicate kinesin-1 motors on the microtubule surface. Scale bars, 50 nm. **E–G**) Side and minus end views of 3D helical reconstructions of vitrified microtubules. Visible density thresholds have been adjusted to levels comparable to docked αβ-tubulin. All maps have been low-pass filtered to 22Å resolution. **E**) Control microtubule without Fab labeling. **F**) Cross section of acetylated microtubule decorated with 6-11B-1 Fab (orange). The structure of the αβ-tubulin dimer [30] has been docked into the right side of the density map (α-tubulin is shown in teal, β-tubulin is shown in purple). **G**) Cross section of deacetylated microtubule decorated with 6-11B-1 Fab (orange).

To generate highly acetylated or completely deacetylated α-tubulins, purified bovine brain tubulin was treated with recombinant MEC-17 or SIRT2 enzymes, respectively, as described [23], [24], [26]. Treatment with MEC-17 resulted in increased levels of acetyl-K40 whereas treatment with SIRT2 resulted in a complete loss of acetyl-K40 signal as determined by western blotting with both monoclonal (6-11B-1) and polyclonal (anti-acetyl-K40) antibodies (Figure 1B, lanes 2 and 3). Both acetylated and deacetylated tubulins polymerized into microtubules with no observable differences in polymerization dynamics or morphology as compared to microtubules polymerized from untreated purified brain tubulin (Figures 2, 4 and data not shown), consistent with previous reports [8], [24], [26], [30]. These results confirm the generation of highly acetylated and completely deacetylated α-tubulin suitable for cryo-EM.

### 2D and 3D EM visualization of the 6-11B-1 Fab within the lumen of microtubules

To probe for the positioning of acetyl-K40 within the microtubule architecture, we generated Fab fragments of the monoclonal antibody 6-11B-1 (Figure S1C) and used them to label microtubules polymerized from highly acetylated (MEC-17-treated) tubulins. In a first step, we examined the labeled microtubules by negative stain EM and observed additional densities bound on the filaments (Figure 2B) as compared to unlabeled untreated microtubules (Figure 2A). The Fab-generated densities appeared along the interior of the filaments but not on the outer edges, unlike kinesin-1 which generates characteristic motor density projections along the filament edges (Figure 2D), giving the first indication that the Fab fragments were localizing in the microtubule lumen. To confirm this observation and directly determine the Fab fragment binding site, we visualized Fab-decorated acetylated microtubules by cryo-EM (Figure S2). The power spectra revealed an additional layerline at 1/8 nm (Figure S3B), indicative of regular Fab decoration with 8 nm spacing. 3D helical reconstructions of selected Fab-decorated acetylated microtubules showed significant density within the microtubule lumen (Figure 2F) that is not observed in unlabeled microtubules (Figure 2E) suggesting that these densities correspond to the Fab fragments. The Fab density follows a helical path with an 8 nm pitch (Figure 2F), revealing attachment to a single α-tubulin subunit of the αβ-tubulin heterodimer.

As a control, we employed cryo-EM and helical reconstructions of microtubules polymerized from completely deacetylated tubulins (SIRT2-treated) and decorated with the same preparation of Fab fragments. Surprisingly, similar densities with the same 8 nm spacing, attributed to Fab, were observed in the lumen of the deacetylated microtubules by both negative stain (Figure 2C) and cryo-EM (Figure 2G). The Fab bound to deacetylated microtubules at lower occupancy than that observed for the acetylated microtubule reconstruction, presumably due to a decreased affinity and/or the presence of unacetylated subunits in the deacetylated tubulin population. Because of the lower Fab occupancy, the corresponding densities appear smaller when shown at comparable thresholds to the 3D maps of Fab-labeled acetylated microtubules. However, adjusting the visible density threshold to lower cut-off levels reveals that the Fab densities establish a connectivity to tubulin that is similar to that observed in the case of acetylated microtubules (data not shown). These results provide the first definitive demonstration that the 6-11B-1 Fab fragment recognizes a K40 epitope that is localized in the microtubule lumen.

### Luminal K40-acetylation does not influence kinesin-1 binding on the microtubule surface

**Figure 3:**
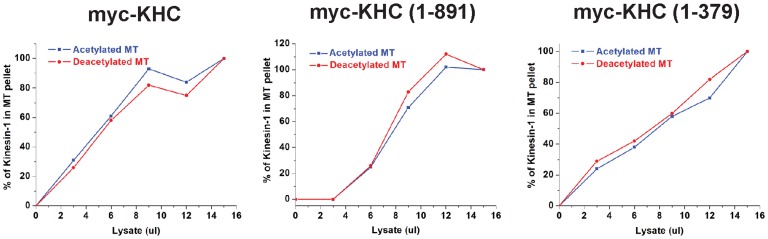
K40 acetylation does not directly influence the binding of Kinesin-1 to microtubules. Myc-tagged versions of full-length kinesin-1 heavy chain (myc-KHC) or truncated, constitutively active constructs (1–891 and 1–379) were expressed in COS7 cells. Increasing amounts of cell lysates were used in microtubule binding assays with a constant amount of taxol-stabilized acetylated (blue lines) or deacetylated (red lines) microtubules and AMPPNP. The percentage of kinesin-1 motor copelleting with microtubules was quantified. Graphs indicate the average of four independent experiments.

The localization of the K40 acetylation site in the microtubule lumen raises the important question of whether a luminal modification can directly influence motors and MAPs on the microtubule surface. The use of MEC-17 and SIRT2 enzymes to generate tubulin species that differ only in the K40 acetylation state allowed us to address this question with respect to the interaction of kinesin-1 motors with microtubules. When constitutively active forms of kinesin-1 motors were mixed with acetylated or deacetylated microtubules in the presence of AMPPNP (a non-hydrolyzable analogue of ATP), no differences were observed in the ability of the kinesin-1 motors to cosediment with acetylated or deacetylated microtubules (Figure 3). These results indicate that the single K40 modification in the microtubule lumen is not sufficient to alter the binding of kinesin-1 to the microtubule surface.

### The 6-11B-1 antibody recognizes both acetylated and deacetylated microtubules

**Figure 4:**
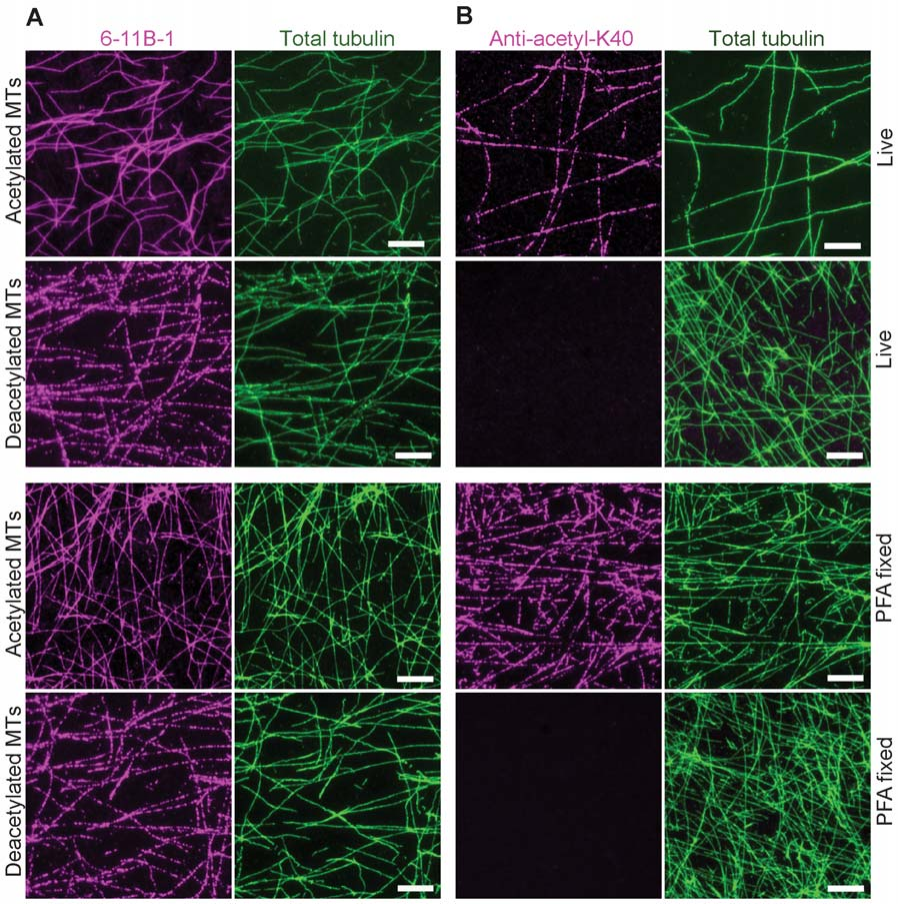
Monoclonal (6-11B-1) and polyclonal (anti-acetyl-K40) antibodies differ in their ability to recognize deacetylated microtubules *in vitro*. Taxol-stabilized microtubules polymerized from acetylated or deacetylated tubulins were stained immediately (Live) or after fixation with paraformaldehyde (PFA fixed) with A) monoclonal 6-11B-1 antibody (magenta) or B) polyclonal anti-acetyl-K40 antibody (magenta). The total tubulin in each sample was detected with DM1A antibody (green). Scale bars, 20 µm.

The fact that the 6-11B-1 Fab fragment decorated both acetylated and deacetylated microtubules was not anticipated. The 6-11B-1 antibody has been widely used to identify and localize acetyl-K40 α-tubulin in a wide variety of animal cells and has been shown to be sensitive to the addition (via MEC-17) or removal (via HDAC6 or SIRT-2) of the acetyl group specifically at K40 [8], [23], [24], [26]. Thus, we tested whether the Fab fragment differs from the whole antibody in its ability to distinguish between acetylated and deacetylated microtubules. To do this, we immunolabeled taxol-stabilized microtubules polymerized from acetylated or deacetylated tubulins with the monoclonal 6-11B-1 and polyclonal anti-acetyl-K40 antibodies. To preclude any effects on antigen recognition by fixation [19], [31], antibodies were added either without fixation (“live”) or after paraformaldehyde fixation (“PFA fixed”) of the microtubules. The monoclonal 6-11B-1 antibody stained both acetylated and deacetylated microtubules regardless of fixation conditions (Figure 4A). In contrast, the polyclonal anti-acetyl-K40 antibody stained acetylated but not deacetylated microtubules (Figure 4B). These results indicate that the monoclonal 6-11B-1 antibody recognizes both acetylated and deacetylated K40 residues within the microtubule polymer.

**Figure 5:**
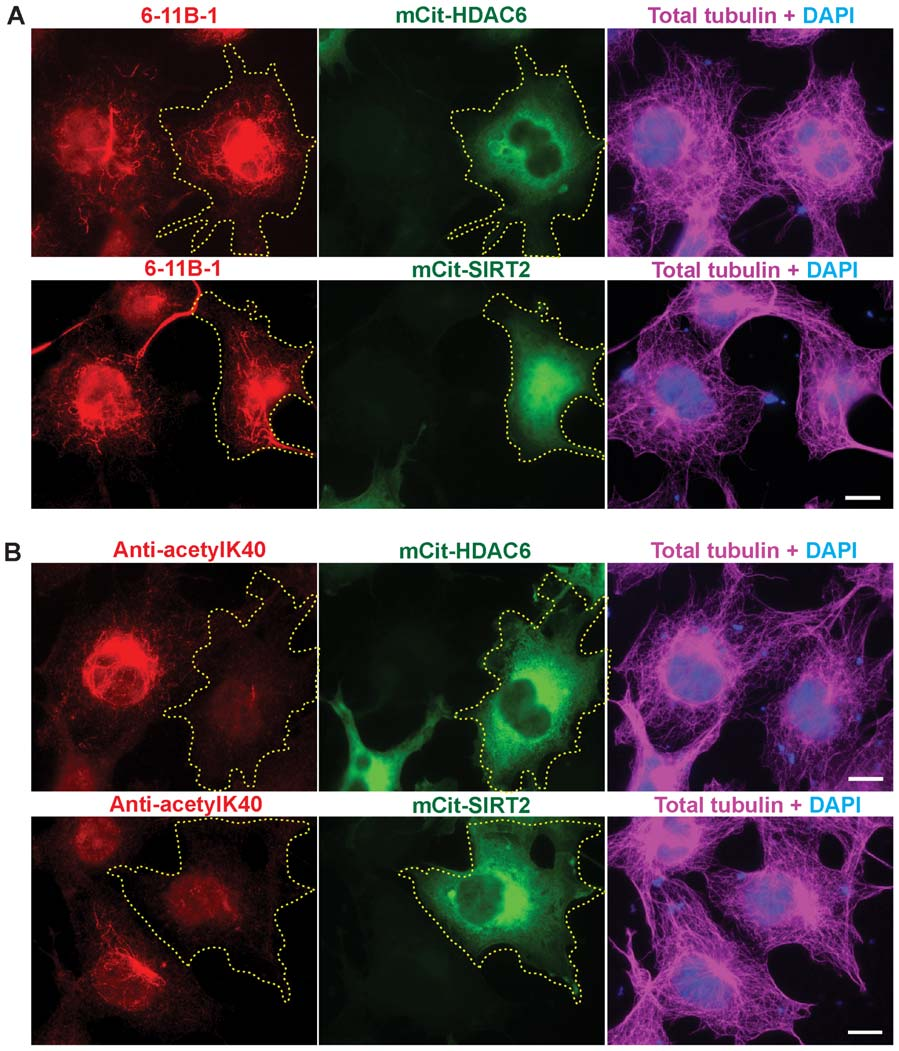
Monoclonal (6-11B-1) and polyclonal (anti-acetyl-K40) antibodies differ in their ability to recognize deacetylated microtubules in cells. COS7 cells expressing the deacetylases mCit-HDAC6 or mCit-SIRT2 (green) were fixed and double stained using A) monoclonal 6-11B-1 (red) and total tubulin (magenta) antibodies or B) polyclonal anti-acetyl-K40 (red) and total tubulin (magenta) antibodies. Transfected cells are indicated by the yellow dotted outline. Scale bars, 20 µm.

To further examine the binding specificities of the monoclonal and polyclonal antibodies, we compared their abilities to recognize acetylated, deacetylated and unacetylated (never modified) α-tubulin subunits in cellular microtubules. Both antibodies failed to label any microtubule structures in PtK2 cells (Figure S4A), indicating that neither antibody recognizes unacetylated K40 residues. Both antibodies labeled highly acetylated microtubules induced by expression of MEC-17 in PtK2 and COS-7 cells (Figure S4B), indicating that both antibodies recognize K40-acetylated microtubules in cells. However, differences were observed in the abilities of the antibodies to recognize deacetylated microtubules in cells. Whereas the polyclonal anti-acetyl-K40 antibody failed to label microtubule structures in cells expressing moderate levels of the K40-deacetyases HDAC6 or SIRT2 (Figure 5B), the monoclonal 6-11B-1 antibody still recognized a large number of cytoplasmic microtubules in expressing cells (Figure 5A; see also Figure S5). Expression of HDAC6 or SIRT2 enzymes does not create an eptiope for 6-11B-1 labeling as the antibody failed to label unacetylated microtubules in PtK2 cells that had been “deacetylated” by expression the deacetylase enzymes (Figure S6). Taken together, the results of Figures 2, 4 and 5 demonstrate that the difference between the antibodies is in binding to deacetylated α-tubulin subunits within microtubules.

## Discussion

These results provide the first definitive demonstration that the K40 acetylation site of α-tubulin is located in the microtubule lumen. This result has important implications for the targeting of the K40 residue by cytoplasmic acetyltransferase and deacetylase enzymes. Since acetylation occurs after polymerization of microtubules in cells [32], [33], our findings indicate that K40-modifying enzymes must access K40 residues present in the microtubule lumen rather than targeting the K40-containing loop from the outside of the microtubule. How do acetyltransferase and deacetylase enzymes access K40 residues in the lumen of the microtubule? One possibility is that the enzymes copolymerize with tubulins and thus reside in the interior of the microtubules. Indeed, cellular microtubules have been found to contain electron scattering material within their lumens [34]. A second possibility is that enzymes enter post-polymerization via the open plus end of the microtubule and diffuse within the microtubule [23], a scenario unlikely based on luminal diffusion rates [35] and the fact that cytoplasmic microtubules are primarily acetylated at internal sites rather than at the open plus ends most accessible to cytoplasmic enzymes [33], [36]. A third possibility is that enzymes access the microtubule lumen post-polymerization via lateral defects that allow “breathing” within the microtubule lattice and/or exchange of tubulin subunits [12], [24], [37]–[39].

Our finding that the K40 acetylation site is located in the microtubule lumen also has important implications for how α-tubulin acetylation can influence events on the microtubule surface. It seems unlikely that the presence or absence of an acetyl group on the luminal K40 residue directly influences motors and MAPs on the microtubule surface. Indeed, we find that for kinesin-1, altering K40 acetylation alone has no effect on the ability of the motor to bind strongly to microtubule. In a similar study, Walter et al. recently showed that K40 acetylation does not directly affect kinesin-1 velocity and run length along the microtubule surface [40]. It thus appears that the influence of K40 acetylation on motor-dependent transport events [15]–[18] is due to additional factors and/or modifications within K40-marked cellular microtubules that are not replicated by alteration of only the K40 acetylation state *in vitro*. For example, K40 acetylation may cause a conformational change in tubulin structure within cellular microtubules or it could serve as a priming event for further tubulin modifications such as additional acetylation events or other PTMs. Further identification of tubulin PTMs and characterization of their effects alone and in concert will be required to test these possibilities.

Both the monoclonal and polyclonal acetyl-K40 antibodies labeled cytoplasmic microtubules in a non-uniform manner (Figure S4A) [33], [36]. The fact that two different antibodies generate similar staining patterns suggests that the non-uniform acetyl-K40 levels along the microtubule filament are not an artifact of antigen recognition by the monoclonal antibody. Rather, it appears that sections of microtubules must differ in their ability to be acetylated at the luminal K40 residue. This difference could be due to spatially restricted accessibility of the K40 residue. Yet the fact that K40 acetylation of polymerized microtubules can be increased by taxol treatment or overexpression of the acetyltransferase MEC-17 [23], [24], [36] suggests that the majority of K40 residues are available for acetylation, at least on a time scale of hours to days. Alternatively, the non-uniform acetyl-K40 levels could be due to local regulation of acetyltransferase and deacetylase activities. Regulation of both MEC-17 and HDAC6 activities has been reported [24], [41]–[44] although spatially restricted regulation of enzymatic activity has not been demonstrated.

These results provide the first demonstration that the monoclonal 6-11B-1 antibody, widely believed to be specific for acetyl-K40 α-tubulin, recognizes both acetylated and deacetylated K40 residues of α-tubulin within the microtubule polymer. Previous work suggested that 6-11B-1 is specific for acetylated α-tubulin based on immunoblotting experiments where cytoplasmic α-tubulin could only be recognized by the antibody after chemical acetylation with acetic anhydride [5], [36]. We now show that the 6-11B-1 antibody also recognizes deacetylated residues within the polymer. We suggest that caution must be taken when interpreting immunostaining results using this antibody. In practical terms, there appears to be little concern about immunostaining normal cycling cells as the 6-11B-1 and anti-acetyl-K40 antibodies recognizes the same acetylated α-tubulin subunits in the spindle, axonemal and cytoplasmic microtubules (data not shown). Caution is urged when immunostaining cells subjected to treatments that appear to alter the levels of acetylated α-tubulin. In these cases, the acetyl-K40 levels must be verified by immunoblotting cell lysates.

We hypothesize that the differences in epitope recognition between the monoclonal 6-11B-1 and polyclonal anti-acetyl-K40 antibodies is due to structural changes in the K40-containing loop. We propose that acetylation causes a conformational change within the K40-containing luminal loop that remains intact after a deacetylation event. That is, the acetylated and deacetylated states of α-tubulin are structurally different than that of unacetylated α-tubulin. We postulate that the polyclonal anti-acetyl-K40 antibody is sensitive to the acetylation state of the K40 residue regardless of the loop conformation whereas the monoclonal 6-11B-1 antibody recognizes the structurally distinct state of acetylated and deacetylated α-tubulin in native microtubules. A structurally distinct state for the K40-containing loop could have important functional consequences on microtubule stability, bending, and interactions. In support of this, differences in lateral protofilament interactions between acetylated and unacetylated microtubules *in vivo* were recently reported [12], [13]. Higher resolution cryo-EM studies of unacetylated, acetylated and deacetylated tubulins may help to reveal the structural consequences of this and other modifications.

## Materials and Methods

### Antibodies and plasmids

Polyclonal antibody production was carried out by ProteinTech Group and the entire study was approved by their Institutional Animal Care and Use Committee (IACUC). All animals were observed on a regular basis for tissue necrosis and abscess formation at the inoculation sites and for the animal's activity, food consumption and body condition. Euthanasia was done under anesthetics with ether with cardiac puncture. Rabbits were immunized with a synthetic peptide (amino acids QMPSD[AcK]TIGG common to all mouse α-tubulin isotypes) coupled to keyhole limpet hemocyanin and boosted at separate locations with the same peptide coupled to BSA. Production bleeds were obtained from the ear vein of sedated rabbits with a 21 gauge needle. Specific antibodies were affinity purified by adsorption to the same peptide coupled to a Sulfolink column (Pierce).

The following monoclonal antibodies were purchased: anti-acetylated tubulin clone 6-11B-1 ([5] Sigma T7451), anti-α-tubulin clone DM1A (Sigma T6199), and anti-β-tubulin clone E7 (Developmental Studies Hybridoma Bank). Secondary antibodies conjugated to fluorophores were purchased from Jackson ImmunoResearch Laboratories. Plasmids for expression of GST-MEC-17 ([23], gift of Jacek Gaertig, University of Georgia), pHEX-His-SIRT2 ([26], gift of Eric Verdin, UCSF) and HA-HDAC6 ([45], gift of Xiang-Jiao Yang, McGill University) have been described. MEC-17, HDAC6 and SIRT2 were sub-cloned by PCR into pmCitrine-C1 for mammalian expression.

### Mammalian cell culture and Immunofluorescence

COS7 (monkey kidney fibroblast, ATCC) cells were grown in DMEM+10% fetal bovine serum (FBS) and 2 mM L-glutamine at 37°C with 5% CO_2_. PtK2 (rat kangaroo kidney epithelial, ATCC) cells were grown in EMEM+10% FBS and 2 mM L-glutamine at 37°C with 5% CO_2_. COS7 and PtK2 cells were transfected using Expressfect (Danville Scientific) and TransIT-LT1 (Mirus-Bio), respectively. The next day, coverslips were rinsed with PBS, fixed in 4% paraformaldehyde in PBS for 10 min, quenched with 50 mM ammonium chloride in PBS for 5 min, and permeabilized in 0.2% Triton-X-100 in PBS for 5 min. Subsequently, coverslips were blocked with 0.2% FSG (Fish Skin Gelatin) in PBS for 5 min and incubated in primary antibody for 1 h. After three washes with 0.2% FSG in PBS, cells were incubated in secondary antibody for 1 h, washed three times in PBS, and mounted with Prolong Gold. Images were obtained on an inverted epifluorescence microscope Nikon TE2000E, equipped with 60X 1.40 NA objective and a Photometrics CoolSnap HQ camera.

### Enzyme purification

His-tagged human SIRT2 protein was bacterially expressed in BL21 (DE3) cells by inducing with 1 mM IPTG (isopropyl-β-D-thiogalactopyranoside) at 37°C for 3 h and purified under native conditions at 4°C by Ni-NTA (Qiagen) as described [26]. GST-tagged human MEC-17 was bacterially expressed in *Rosetta2* cells, adsorbed to Glutathione Sepharose beads (GE Healthcare Biosciences), and eluted with in 50 mM Tris-HCl pH-8.0, 0.2 mM EDTA, 10 mM reduced glutathione as described. Purified proteins were dialyzed against dialysis buffer (20 mM Tris-HCl pH-8.0, 0.2 mM DTT) overnight at 4°C, mixed with 10% glycerol, and snap frozen in liquid nitrogen prior to storage at −80°C.

### Tubulin modification and polymerization

Purified bovine brain tubulin was purchased from Cytoskeleton (TL238). Acetylated tubulin was generated by incubating purified bovine brain tubulin with recombinant GST-MEC-17 in the presence of 10 µM Acetyl coenzyme A (Sigma A2056) for 2 h at 28°C with constant agitation. Deacetylated tubulin was prepared by incubating purified bovine brain tubulin with recombinant His-SIRT2 in the presence of 1 mM NAD (β-Nicotinamide adenine dinucleotide, Sigma N8285) for 2 h at 37°C with constant mixing. The resulting modified tubulins were cycled through one round of polymerization/depolymerization to remove the enzyme (verified by SDS-PAGE, data not shown) before flash-freezing in liquid nitrogen and storage at −80°C. Untreated, acetylated or deacetylated tubulins were polymerized for 20 min at 37°C in BRB80 at a concentration of 10 mg/ml in the presence of 20% DMSO (v/v), 2 mM GTP, 20 µM taxol, 0.5 mM PMSF and 4 mM MgCl_2_.

### Kinesin binding assay

Constitutively active rat kinesin heavy chain (KIF5C) constructs were expressed in COS-7 cells. The cells were lysed in lysis buffer (25 mM HEPES/KOH pH-7.4, 115 mM KOAc, 5 mM NaOAc, 5 mM MgCl_2_, 0.5 mM EGTA and 1% Triton X-100) containing 0.1 mM ATP and clarified by centrifugation at 14,000 rpm for 10 min at 4°C. Taxol-stabilized acetylated or deacetylated microtubules were added to 0.1 mg/ml together with 20 µM taxol and 1 mM AMPPNP (a non-hydrolyzable ATP analog) and incubated for 30 min at room temperature with constant mixing. The motor-microtubule complexes were sedimented at 90,000 rpm at 18°C for 10 min through a glycerol cushion (BRB80 containing 60% glycerol and 20 µM taxol). The pellet was dissolved in SDS-PAGE sample buffer and the amount of tubulins and motors in the pellets was determined by immunoblotting. The scanned blots were used for quantification in ImageJ software.

### Fab fragment preparation and microtubule decoration

Fab fragments of 6-11B-1 antibodies were generated using the Fab micro-preparation kit (Pierce). In brief, 6-11B-1 IgG was digested with immobilized papain at 37°C for 6 h with end-over-end mixing. The digested products were eluted by low speed centrifugation and the Fc fragments were bound to protein A beads at room temperature for 20 min. Fab fragments were collected by low speed spin and concentrated.

Untreated microtubules were incubated with GST-KHC motor domain (KR01, Cytoskeleton), whereas acetylated or deacetylated microtubules were incubated with 6-11B-1 Fab fragment at a 1:2 ratio for 1 h at room temperature. Excess motors and Fab fragments were removed by centrifugation of microtubules through a glycerol cushion (BRB80 containing 60% glycerol and 20 µM taxol) in a TLA100 rotor at 90,000 rpm for 10 min at 35°C. Fab-decorated microtubules were resuspended in BRB80 containing 20 µM taxol.

### Electron Microscopy and 3D Reconstruction

Samples were prepared for negative stain electron microscopy using 0.75% solution of uranyl formate and conventional negative staining protocols [46]. For cryo-EM, 2 µl of the microtubule samples were applied on glow-discharged Quantifoil R2/2 200 holey carbon grids and vitrified using a Vitrobot Mark IV (FEI Co.). Vitrified specimen was imaged on a Tecnai F20 Transmission Electron Microscope (FEI Co.) equipped with a field emission gun and operated at 200 kV. Images were recorded at a magnification of 66,964x on a Gatan US4000 CCD camera at a ∼2 µm defocus. The pixel size of images acquired under these conditions is 2.24 Å.

Micrographs were screened for helical, 15-protofilament microtubules using the PHOELIX software package [47]. For 3D reconstructions of microtubule-Fab complexes, we selected filaments with strong signal at the 1/8 nm layerline in the 2D power spectra, indicative of high levels of Fab decoration. For the 3D reconstruction of the acetylated microtubule in complex with Fab, we selected and averaged layer-line data from 42 near and far sides. For the 3D recontruction of deacetylated with attached Fab and control microtubules, we selected and averaged layer-line data from 10 near and far sides.

### Immunostaining of acetylated and deacetylated microtubules

Polymerized microtubules were adsorbed onto coverslips and stained with 6-11B-1 antibodies without fixation (live) or after fixation with 4% paraformaldehyde (PFA fixed) in PBS containing 20 µM taxol. All subsequent steps were carried out in BRB80+20 µM taxol. The cover slips were blocked with 5 mg/ml casein for 30 min, incubated with primary antibodies for 1 h, washed three times, incubated with secondary antibodies for 1 h, washed three times, and mounted with Prolong Gold. The images were obtained on an inverted epi-fluorescence microscope Nikon TE2000E, equipped with 60X 1.40 NA objective and a Photometrics CoolSnap HQ camera.

## Supporting Information

Figure S1 **Purification of recombinant MEC-17 and SIRT2 enzymes and Fab fragment preparation.** **A,B)** Coomassie-stained SDS-PAGE gels showing purification profile of recombinant A) GST-MEC-17 or B) His-SIRT2. **C)** Coomassie-stained SDS-PAGE gel showing preparation of Fab fragments from the monoclonal 6-11B-1 antibody.
(TIF)

Figure S2 **Raw cryo-EM images of representative microtubule segments.** Filament sections have been excised from larger micrographs and enlarged to show detail. Shown are representative sections of A) control (no enzyme treatment, no Fab binding), B) MEC-17-acetylated and 6-11B-1 Fab-decorated, and C) SIRT2-deactylated and 6-11B-1 Fab-decorated microtubules. Scale bar, 25 nm.
(TIF)

Figure S3 **Representative power spectra.** **A)** A representative power spectrum from a single vitrified control microtubule. **B)** A representative power spectrum from a single vitrified MEC-17-acetylated microtubule decorated with 6-11B-1 Fab. Regular Fab decoration is indicated by the presence of a 1/8 nm layer line, compared to the control microtubule (A). **C)** A representative power spectrum from a single vitrified SIRT2-deacetylated microtubule decorated with 6-11B-1 Fab. A weaker 1/8 nm signal is observed, corresponding to lower Fab occupancy.
(TIF)

Figure S4 **Monoclonal 6-11B-1 and polyclonal anti-acetyl-K40 antibodies recognize acetylated but not unacetylated microtubules in cells.** **A)** COS7 and PtK2 cells were fixed and double stained with monoclonal 6-11B-1 and total tubulin antibodies (left panels) or polyclonal anti-acetyl-K40 and total tubulin antibodies (right panels). Neither antibody recognizes microtubule filaments in PtK2 cells which contain only unacetylated (never modified) α-tubulin. **B)** COS7 and PtK2 cells expressing the acetytransferase mCit-MEC-17 (green) were double stained with monoclonal 6-11B-1 (red) and total tubulin (magenta) antibodies (left panels) or with polyclonal anti-acetyl-K40 (red) and total tubulin (magenta) antibodies (right panels). Both antibodies recognize the highly acetylated microtubules induced by expression of mCit-MEC-17. Scale bars, 20 µm.
(TIF)

Figure S5 **The 6-11B-1 epitope is sensitive to the level of expression of the α-tubulin K40 deacetylases HDAC6 and SIRT2.** COS7 cells transfected with A) mCit-HDAC6 or B) mCit-SIRT2 were fixed and stained with monoclonal 6-11B-1 (red) and total tubulin (magenta) antibodies. Scale bars, 20 µm. Transfected cells are indicated by a yellow dotted outline. Previous work showed that expression of HDAC6 or SIRT2 in mammalian cells resulted in a complete loss of 6-11B-1 staining [1]–[3], suggesting that the 6-11B-1 antibody does not recognize deacetylated α-tubulin. In contrast, we show in Figure 4 that moderate expression of HDAC6 or SIRT2 results in deacetylated microtubules that can still be recognized by the 6-11B-1 antibody. To explain the difference between our results and the previous work, we looked at 6-11B-1 and anti-acetyl-K40 labeling at different levels of deacetylase expression. Figure 5 shows cells expressing moderate levels of HDAC6 and SIRT2 expression (based on fluorescence intensity) whereas this figure shows cells expressing high levels of HDAC6 and SIRT2. In agreement with previous work [1]–[3], this figure shows that 6-11B-1 antigenicity is lost in cells expressing high levels of HDAC6 or SIRT2 enzymes. The fact that the polyclonal anti-acetyl-K40 antibody does not recognize any microtubules even in cells expressing moderate levels of HDAC6 or SIRT2 enzymes (Figures 5B), indicates that expression of these deacetylase enzymes results in microtubules that are fully non-acetylated (deacetylated and unacetylated). The fact that 6-11B-1 stains microtubules in cells expressing moderate levels of HDAC6 or SIRT2 (Figure 5A) but not high levels of the enzymes (Figure S5) indicates that α-tubulin subunits undergo a structural conversion from the deacetylated (recognized by 6-11B-1) to non-acetylated (not recognized by 6-11B-1) state. Whether this conversion is due to increased levels or time of deacetylase expression is presently unclear. 1. North BJ, Marshall BL, Borra MT, Denu JM, Verdin E (2003) The human Sir2 ortholog, SIRT2, is an NAD(+)-dependent tubulin deacetylase. Mol Cell 11: 437-444. 2. Matsuyama A, Shimazu T, Sumida Y, Saito A, Yoshimatsu Y, et al. (2002) In vivo destabilization of dynamic microtubules by HDAC6-mediated deacetylation. EMBO J 21: 6820–6831. 3. Zhang Y, Li N, Caron C, Matthias G, Hess D, et al. (2003) HDAC-6 interacts with and deacetylates tubulin and microtubules in vivo. EMBO J 22: 1168–1179.
(TIF)

Figure S6 **HDAC6 or SIRT2 binding does not create an epitope for the 6-11B-1 antibody in PtK2 cells.** PtK2 cells expressing the deacetylases mCit-HDAC6 or mCit-SIRT2 (green) were fixed and double stained using monoclonal 6-11B-1 anti-acetylated tubulin (red) and total tubulin (magenta) antibodies. Transfected cells are indicated by the yellow dotted outline. Scale bars, 20 µm.
(TIF)
