# Supplementary figures and images for: Luminal Localization of α-tubulin K40 Acetylation by Cryo-EM Analysis of Fab-Labeled Microtubules
Source: PLoS One. 2012 Oct 26;7(10):e48204. doi: 10.1371/journal.pone.0048204 (PMC3482196; doi:10.1371/journal.pone.0048204)

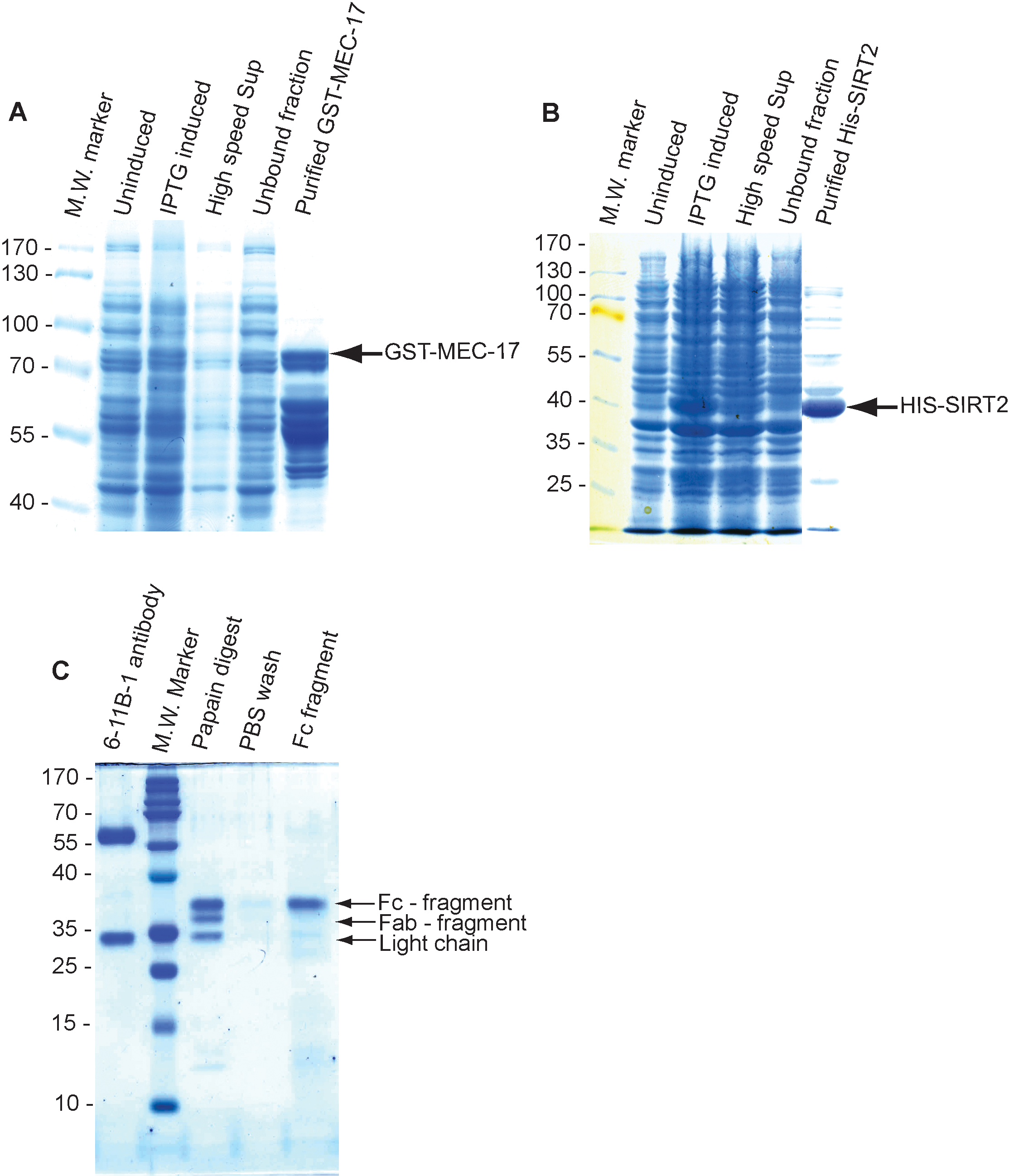

Supplement: Figure S1 — Purification of recombinant MEC-17 and SIRT2 enzymes and Fab fragment preparation. A,B) Coomassie-stained SDS-PAGE gels showing purification profile of recombinant A) GST-MEC-17 or B) His-SIRT2. C) Coomassie-stained SDS-PAGE gel showing preparation of Fab fragments from the monoclonal 6-11B-1 antibody. (TIF) [file pone.0048204.s001.tif]

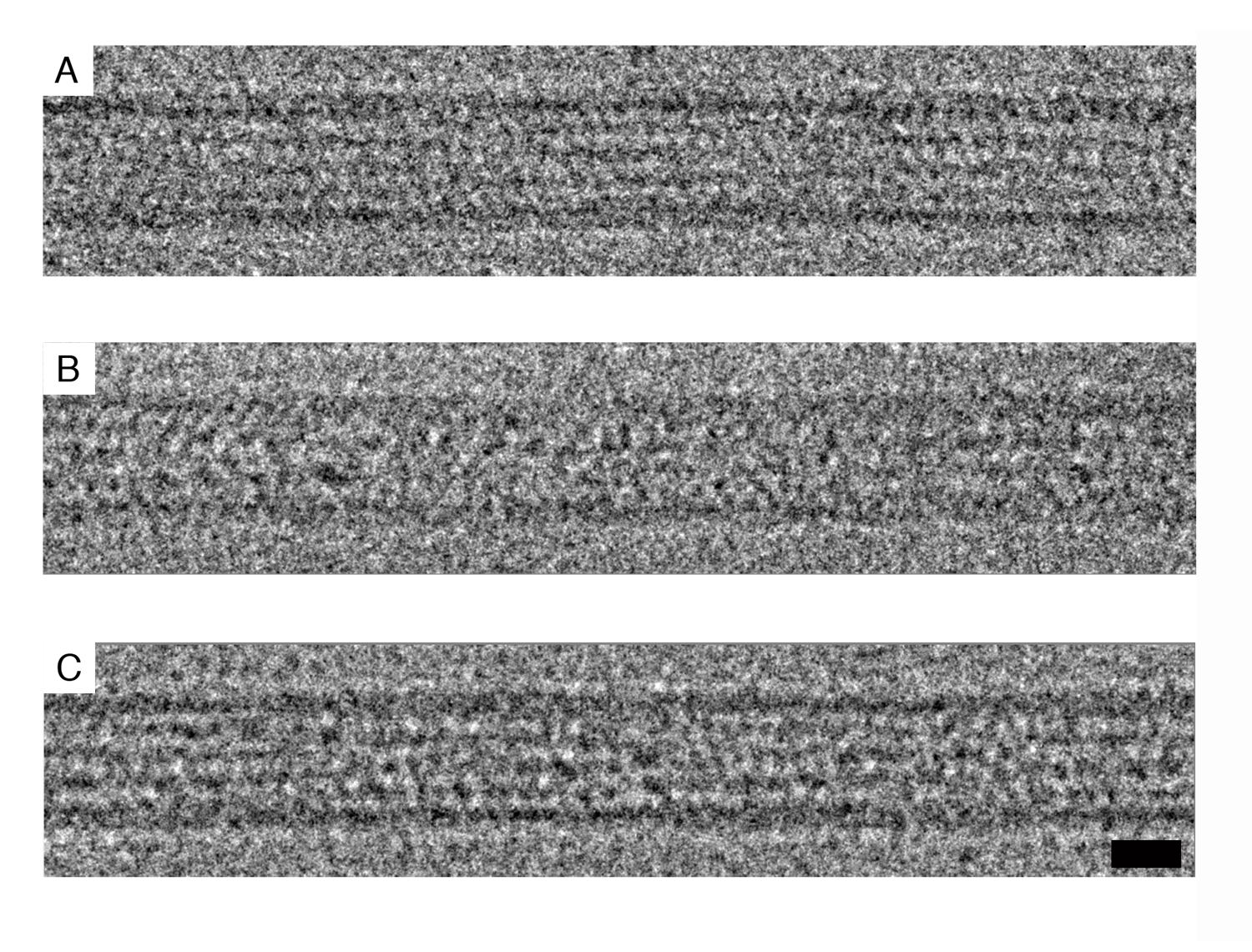

Supplement: Figure S2 — Raw cryo-EM images of representative microtubule segments. Filament sections have been excised from larger micrographs and enlarged to show detail. Shown are representative sections of A) control (no enzyme treatment, no Fab binding), B) MEC-17-acetylated and 6-11B-1 Fab-decorated, and C) SIRT2-deactylated and 6-11B-1 Fab-decorated microtubules. Scale bar, 25 nm. (TIF) [file pone.0048204.s002.tif]

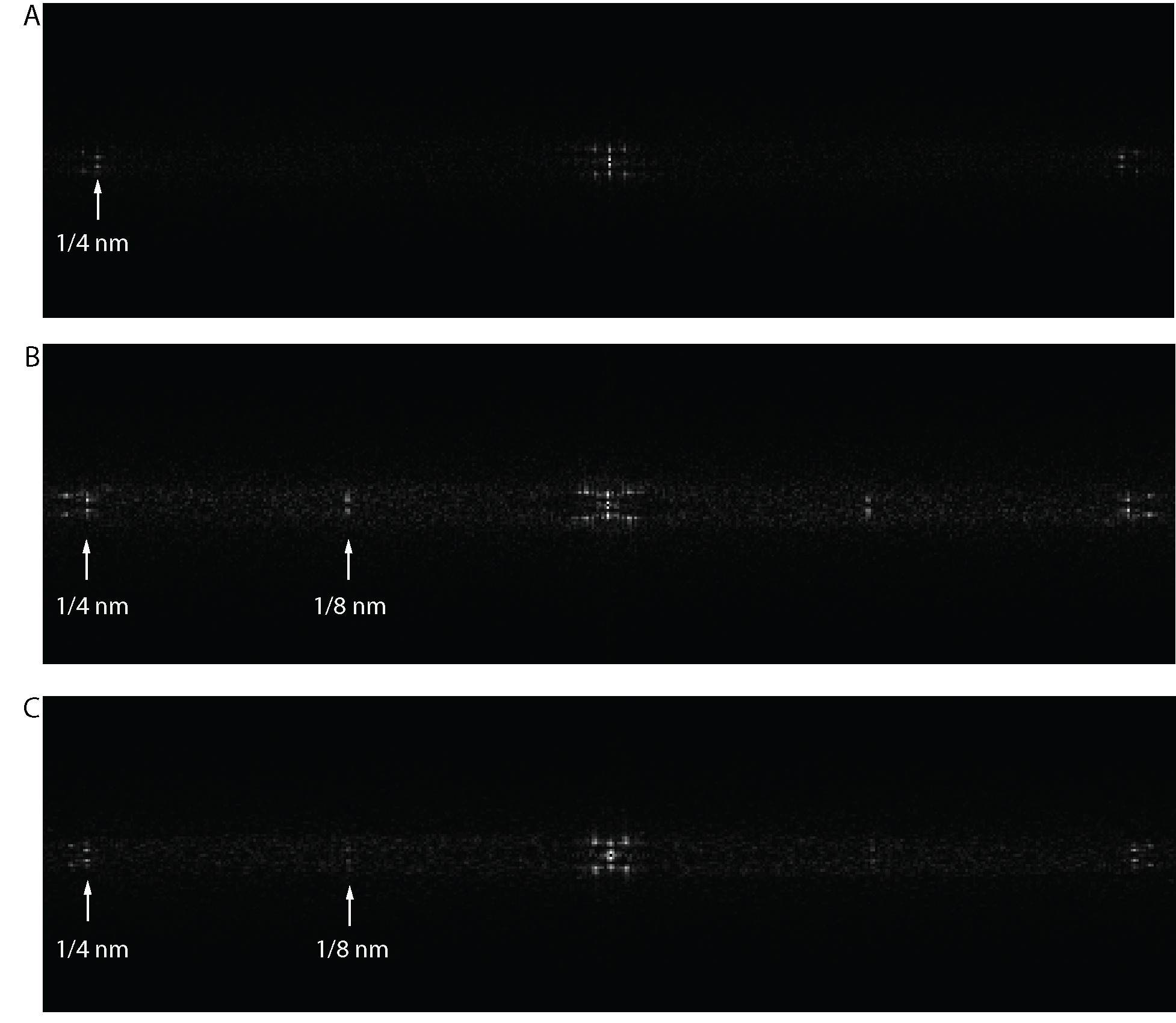

Supplement: Figure S3 — Representative power spectra. A) A representative power spectrum from a single vitrified control microtubule. B) A representative power spectrum from a single vitrified MEC-17-acetylated microtubule decorated with 6-11B-1 Fab. Regular Fab decoration is indicated by the presence of a 1/8 nm layer line, compared to the control microtubule (A). C) A representative power spectrum from a single vitrified SIRT2-deacetylated microtubule decorated with 6-11B-1 Fab. A weaker 1/8 nm signal is observed, corresponding to lower Fab occupancy. (TIF) [file pone.0048204.s003.tif]

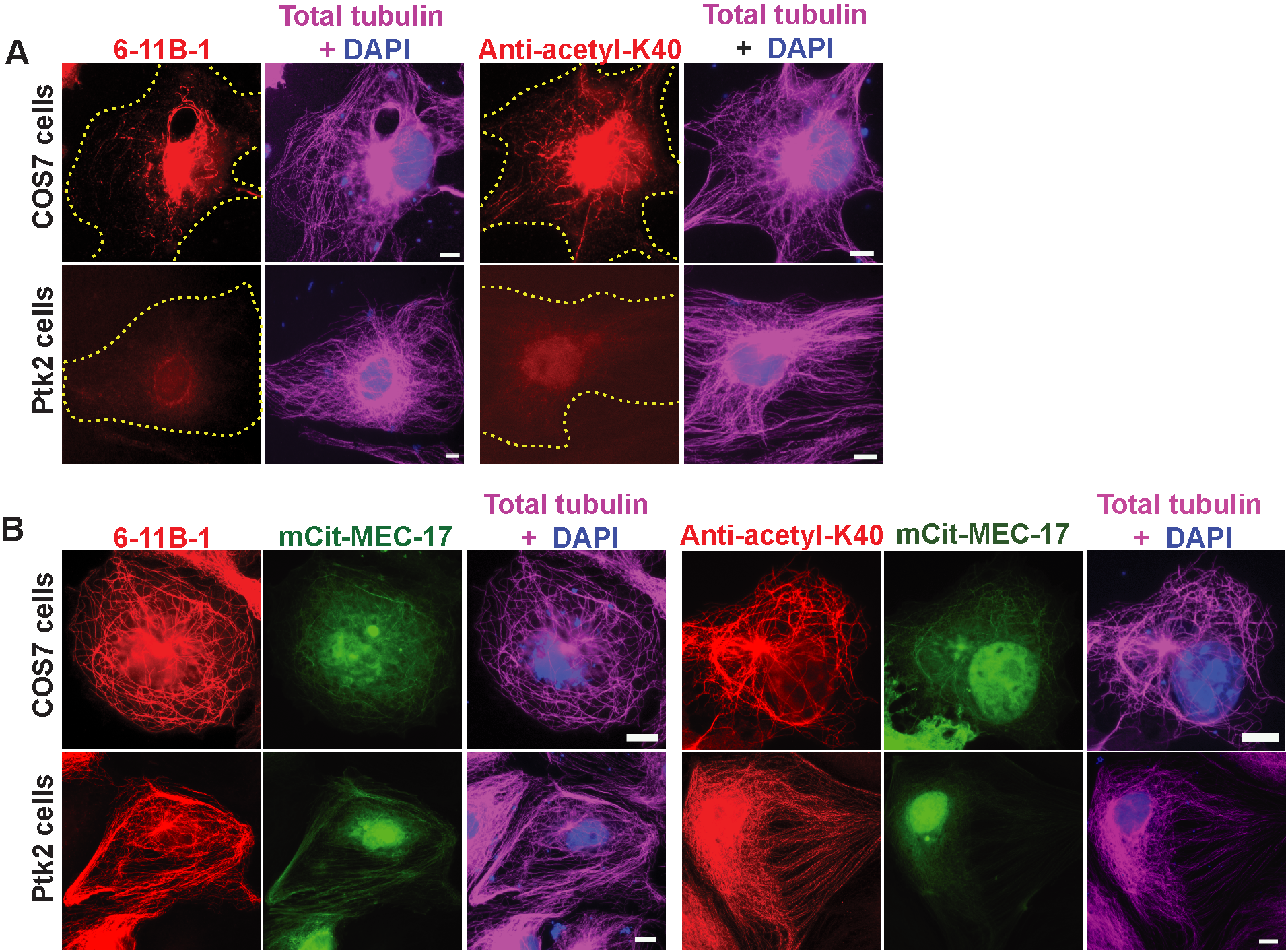

Supplement: Figure S4 — Monoclonal 6-11B-1 and polyclonal anti-acetyl-K40 antibodies recognize acetylated but not unacetylated microtubules in cells. A) COS7 and PtK2 cells were fixed and double stained with monoclonal 6-11B-1 and total tubulin antibodies (left panels) or polyclonal anti-acetyl-K40 and total tubulin antibodies (right panels). Neither antibody recognizes microtubule filaments in PtK2 cells which contain only unacetylated (never modified) α-tubulin. B) COS7 and PtK2 cells expressing the acetytransferase mCit-MEC-17 (green) were double stained with monoclonal 6-11B-1 (red) and total tubulin (magenta) antibodies (left panels) or with polyclonal anti-acetyl-K40 (red) and total tubulin (magenta) antibodies (right panels). Both antibodies recognize the highly acetylated microtubules induced by expression of mCit-MEC-17. Scale bars, 20 µm. (TIF) [file pone.0048204.s004.tif]

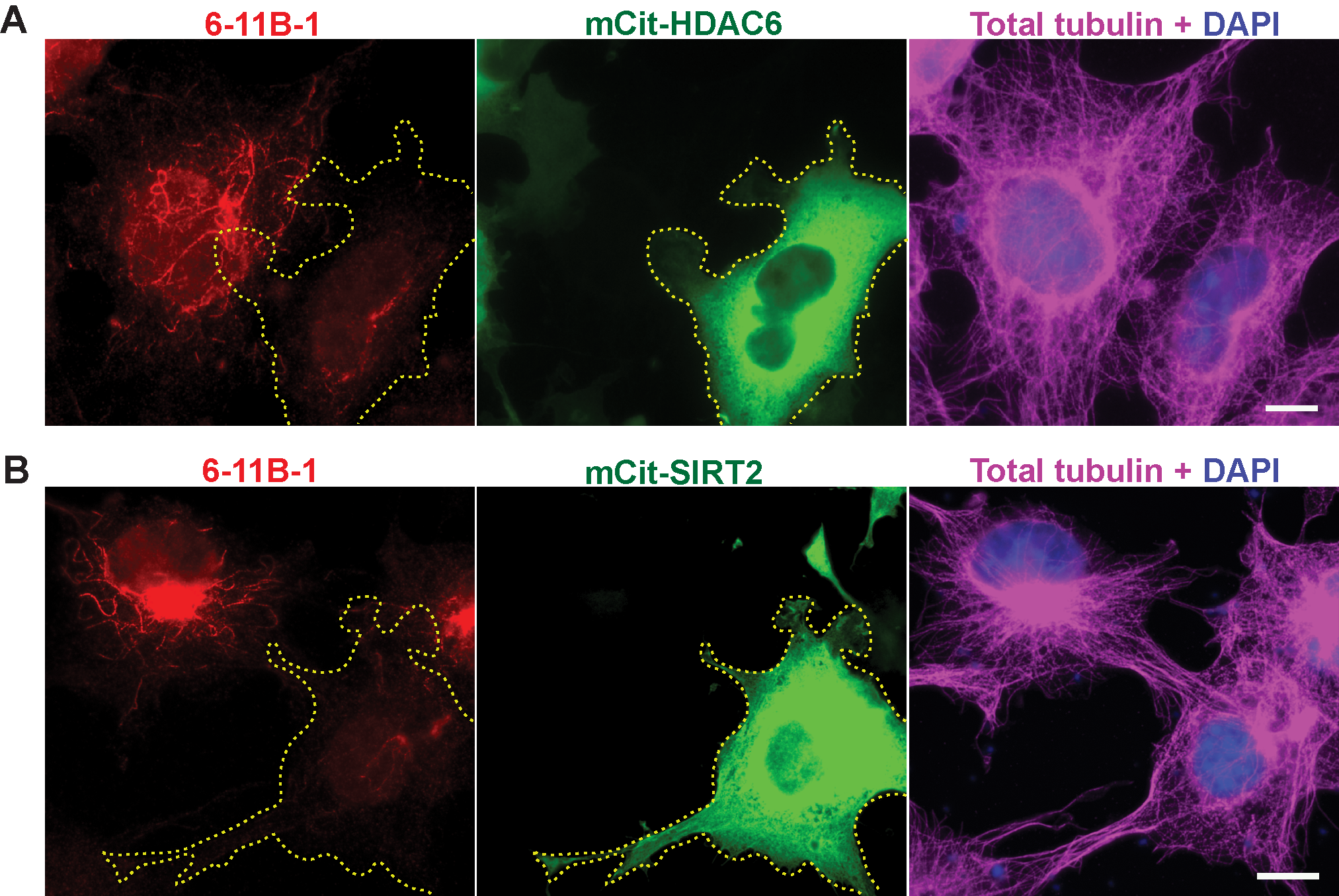

Supplement: Figure S5 — The 6-11B-1 epitope is sensitive to the level of expression of the α-tubulin K40 deacetylases HDAC6 and SIRT2. COS7 cells transfected with A) mCit-HDAC6 or B) mCit-SIRT2 were fixed and stained with monoclonal 6-11B-1 (red) and total tubulin (magenta) antibodies. Scale bars, 20 µm. Transfected cells are indicated by a yellow dotted outline. Previous work showed that expression of HDAC6 or SIRT2 in mammalian cells resulted in a complete loss of 6-11B-1 staining [1]–[3], suggesting that the 6-11B-1 antibody does not recognize deacetylated α-tubulin. In contrast, we show in Figure 4 that moderate expression of HDAC6 or SIRT2 results in deacetylated microtubules that can still be recognized by the 6-11B-1 antibody. To explain the difference between our results and the previous work, we looked at 6-11B-1 and anti-acetyl-K40 labeling at different levels of deacetylase expression. Figure 5 shows cells expressing moderate levels of HDAC6 and SIRT2 expression (based on fluorescence intensity) whereas this figure shows cells expressing high levels of HDAC6 and SIRT2. In agreement with previous work [1]–[3], this figure shows that 6-11B-1 antigenicity is lost in cells expressing high levels of HDAC6 or SIRT2 enzymes. The fact that the polyclonal anti-acetyl-K40 antibody does not recognize any microtubules even in cells expressing moderate levels of HDAC6 or SIRT2 enzymes (Figures 5B), indicates that expression of these deacetylase enzymes results in microtubules that are fully non-acetylated (deacetylated and unacetylated). The fact that 6-11B-1 stains microtubules in cells expressing moderate levels of HDAC6 or SIRT2 (Figure 5A) but not high levels of the enzymes (Figure S5) indicates that α-tubulin subunits undergo a structural conversion from the deacetylated (recognized by 6-11B-1) to non-acetylated (not recognized by 6-11B-1) state. Whether this conversion is due to increased levels or time of deacetylase expression is presently unclear. 1. North BJ, Marshall BL, Borra MT, [file pone.0048204.s005.tif]

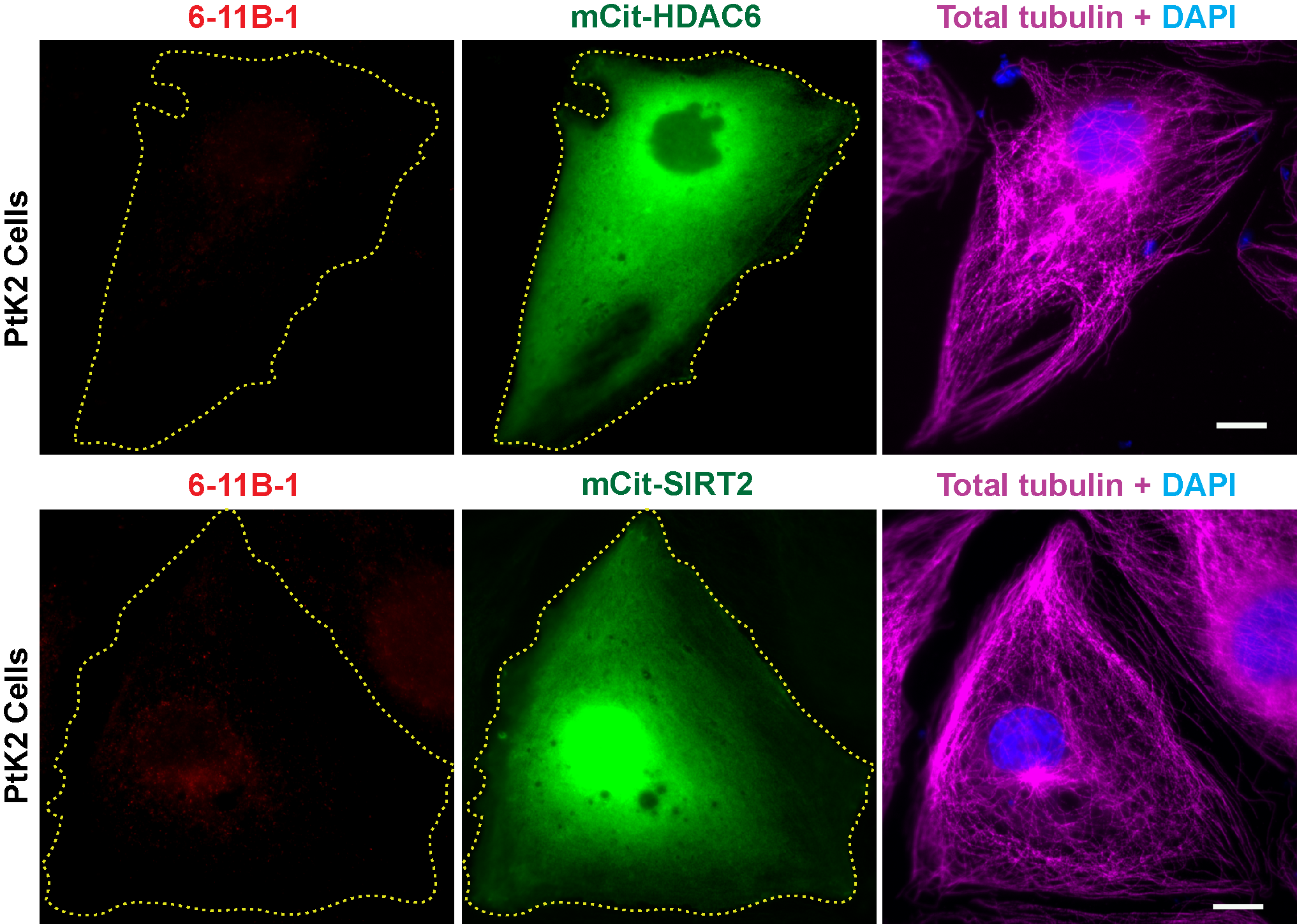

Supplement: Figure S6 — HDAC6 or SIRT2 binding does not create an epitope for the 6-11B-1 antibody in PtK2 cells. PtK2 cells expressing the deacetylases mCit-HDAC6 or mCit-SIRT2 (green) were fixed and double stained using monoclonal 6-11B-1 anti-acetylated tubulin (red) and total tubulin (magenta) antibodies. Transfected cells are indicated by the yellow dotted outline. Scale bars, 20 µm. (TIF) [file pone.0048204.s006.tif]
